# Prognostic Value of Matrix Metalloproteinase 9 (MMP9) in Patients Following Off-Pump Coronary Artery Bypass Grafting

**DOI:** 10.3390/life15060908

**Published:** 2025-06-04

**Authors:** Mikhail Popov, Siarhei Dabravolski, Vladislav Dontsov, Sergei Vzvarov, Evgeniy Agafonov, Dmitriy Zybin, Olga Radchenkova, Dmitriy Saveliev, Victoria Pronina, Natalia Kashirina, Liudmila Lipatova, Mikhail Peklo, Pavel Rutkevich, Elena Yanushevskaya, Alisa Sokolovskaya, Arkadiy Metelkin, Svetlana Verkhova, Nikita Nikiforov, Dmitriy Shumakov

**Affiliations:** 1 M.F. Vladimirsky Moscow Regional Clinical Research Institute, Schepkina St. 61/2, 129110 Moscow, Russia; popovcardio88@mail.ru (M.P.); vvdontsov@yandex.ru (V.D.); sergejvzvarov@gmail.com (S.V.); agafonov.cardiacsurger@mail.ru (E.A.); poison1983@inbox.ru (D.Z.); morgunovaolya@yandex.ru (O.R.); dima_saveliev14072000@mail.ru (D.S.); vpronina85@gmail.com (V.P.); sdvtranspl@rambler.ru (D.S.); 2 Institute of General Pathology and Pathophysiology, Baltiyskaya Str. 8, 125315 Moscow, Russia; alice.sokolovskaya@gmail.com (A.S.); armetelkin@gmail.com (A.M.); verxova.svetlana@gmail.com (S.V.); nikiforov.mipt@googlemail.com (N.N.); 3 Department of Biotechnology Engineering, Braude Academic College of Engineering, Snunit 51, P.O. Box 78, Karmiel 2161002, Israel; 4 National Medical Research Center of Cardiology Named After Academician E.I. Chazov, 21552 Moscow, Russia; kashka55@yandex.ru (N.K.); liliudon3005@yandex.ru (L.L.); peclo@yandex.ru (M.P.); p.rutkevich@gmail.com (P.R.); yanushevskaya@yandex.ru (E.Y.); 5 Laboratory of Cellular and Molecular Pathology of Cardiovascular System, Federal State Budgetary Scientific Institution “Petrovsky National Research Centre of Surgery”, Tsyurupy Str. 3, 117418 Moscow, Russia; 6 Core Facility Center, Institute of Gene Biology, Russian Academy of Sciences, Vavilova Str., 34/5, 119334 Moscow, Russia

**Keywords:** ischaemic heart disease, matrix metalloproteinase 9 (MMP9), biomarker, myocardial revascularisation

## Abstract

Background: Matrix metalloproteinase 9 (MMP9) has recently emerged as a risk predictor in patients with cardiovascular diseases (CVD). However, little is known regarding the significance of elevated plasma MMP9 levels in patients during the long-term period following myocardial revascularisation. We aimed to investigate the role of MMP9 in relation to myocardial status before and after myocardial revascularisation and to assess its long-term prognostic value. Methods: This prospective observational study included 200 male patients with ischaemic heart disease. All patients underwent direct myocardial revascularisation on a beating heart (off-pump surgery). Plasma MMP9 levels were analysed preoperatively, at 48 h postoperatively, and during the long-term follow-up period (one year postoperatively). Key echocardiographic parameters, specifically left ventricular ejection fraction (LVEF) and Left Ventricular End-Diastolic Volume (LVEDV), were also assessed. Results: MMP9 levels decreased significantly at 48 h postoperatively (*p* < 0.0001). During the long-term postoperative period, a clear relationship was demonstrated: higher 1-year MMP9 levels were associated with lower 1-year LVEF, whilst lower 1-year MMP9 levels were associated with higher 1-year LVEF. No significant correlation was observed between preoperative MMP9 levels and age or most other baseline laboratory parameters. Conclusions: Our study established an association between 1-year postoperative MMP9 levels and key parameters of left ventricular function during the long-term follow-up period. This suggests that MMP9 may serve as a novel biomarker for predicting outcomes following myocardial revascularisation.

## 1. Introduction

Increased population morbidity and mortality from cardiovascular diseases (CVD) play a significant role in shaping unfavourable demographic situations worldwide. Society bears substantial human losses and economic burden. Experts predict that mortality from CVD will continue to rise, driven, in no small part, by the gradual increase in life expectancy [1].

Over recent years, ischaemic heart disease (IHD) has remained a leading pathology within the spectrum of CVD in economically developed countries and ranks among the primary causes of population mortality. Key complications of IHD include myocardial infarction (MI) and the subsequent development of chronic heart failure (CHF) resulting from left ventricular (LV) remodelling [2].

At present, the causes and factors contributing to unfavourable outcomes of CHF secondary to IHD, as well as the risks of disease progression, remain poorly understood [3]. Consequently, identifying predictors of long-term LV remodelling progression following myocardial revascularisation in patients with IHD, which can lead to the recurrence of CHF, is crucial [4].

Matrix metalloproteinase 9 (MMP9) is specifically implicated in adverse LV remodelling and participates in acute processes, such as the regulation of vascular tone and platelet aggregation [5]. Moreover, plasma MMP9 levels are significantly elevated during the early period following MI. Myocardial injury is a common complication occurring in the perioperative period and is associated with adverse outcomes, such as higher mortality [6]. In response to ischaemia–reperfusion (I/R), as well as inflammation and oxidative stress, MMP9 expression levels are significantly upregulated [7]. This plasma biomarker appears particularly relevant in the context of coronary artery bypass grafting (CABG) surgery in patients with IHD, a procedure that involves most of the aforementioned mechanisms [8]. However, there is a lack of adequately analysed data on the potential role and impact of MMP9 on long-term outcomes following myocardial revascularisation in patients with IHD [9,10,11,12]. Therefore, we designed the present study to evaluate plasma MMP9 levels and their potential utility in patients with IHD following myocardial revascularisation and to assess their association with clinical cardiac dysfunction.

## 2. Materials and Methods

### 2.1. Patient Population

This study was conducted between 2020 and 2023, enrolling 200 male patients with multivessel coronary artery disease. All patients underwent surgical myocardial revascularisation without cardiopulmonary bypass (off-pump surgery). Patients were excluded if they met one or more of the following criteria: acute MI within the preceding 30 days (given the associated increase in inflammatory markers and acute phase proteins, which directly affect MMP9 levels); CHF New York Heart Association (NYHA) functional class III–IV; malignancy; haematological disorders; age <40 or >70 years; emergency surgery; or cardiomyopathy.

This study was conducted in accordance with the ethical principles of the Declaration of Helsinki (World Medical Association, 1964, revised 2004) and was approved by the Ethics Committee of the Moscow Regional Research and Clinical Institute (MONIKI) (Protocol No. 6, dated 14 May 2019). Written informed consent was obtained from all participants prior to enrolment. All patient data were kept strictly confidential.

The control group consisted of 50 individuals (39 males and 11 females) aged 45–60 years (see Table 1 for baseline characteristics). Individuals were eligible for the control group if they had no history of cardiovascular pathology according to coronary angiography data, no haematological disorders, and no malignancy. Exclusion criteria also included the use of illicit drugs and the use of medications known to affect platelet function. MMP9 levels in the control group were measured immediately after confirmation of eligibility. All data for the study and control groups were collected from medical records.

Blood samples (approximately 5 mL) were obtained from the antecubital vein of participants using disposable vacuum collection tubes. Samples were processed within 30 min of venepuncture. For the patient group, blood samples were collected preoperatively and on the second postoperative day (48 h).

### 2.2. Perioperative Management

In the operating theatre, following the establishment of invasive arterial blood pressure monitoring via the radial artery, general anaesthesia was induced. Patients were intubated and mechanically ventilated with a fraction of inspired oxygen (FiO2) of 40%. After a median sternotomy, the saphenous vein graft (SVG) and the left internal mammary artery (LIMA) were harvested. Coronary artery bypass grafting was performed off-pump, utilising intracoronary shunts and a myocardial stabiliser. A side-biting aortic clamp was used during the construction of proximal anastomoses. Patients were transferred to the Cardiac Intensive Care Unit (CICU) following sternal closure.

### 2.3. Measurement of MMP9 Concentration

Plasma concentrations of MMP9 were determined using a commercial enzyme-linked immunosorbent assay (ELISA) kit (Human MMP-9 Immunoassay, Cat.# DMP900, R&D Systems, Inc., Minneapolis, MN, USA). Whole blood was collected into Vacuette tubes containing lithium heparin and centrifuged at 1000× *g* for 15 min to separate the plasma. The collected plasma was then recentrifuged at 10,000× *g* for 10 min in a refrigerated centrifuge to obtain platelet-poor plasma. For the ELISA, patient plasma samples were diluted 1:40. MMP9 concentrations were calculated using standard calibration curves according to the manufacturer’s instructions.

### 2.4. Statistical Analysis

Data analysis was performed using IBM SPSS Statistics (version 27; IBM Corp, Armonk, NY, USA) and GraphPad Prism version 8.0 (GraphPad Software Inc., La Jolla, CA, USA). The normality of data distribution was assessed using the Shapiro–Wilk test. Given that most continuous data were not normally distributed, they are presented as median and interquartile range (IQR: Q1–Q3) (minimum–maximum values). Categorical variables are presented as percentages (%).

A comprehensive set of statistical methods was employed. Changes in binary outcomes across paired measurements were assessed using McNemar’s test. Associations between categorical variables were examined using Pearson’s chi-square test with Yates’ continuity correction or Fisher’s exact test for small, expected cell counts. The strength of these associations was quantified using the phi (φ) coefficient for 2 × 2 tables and Cramer’s V for larger tables.

Continuous variables were compared using non-parametric tests: the Wilcoxon signed-rank test for paired samples (e.g., preoperative vs. postoperative MMP9 in the patient group) and the Mann–Whitney U test for independent groups (e.g., patient group vs. control group, or high MMP9 subgroup vs. low MMP9 subgroup). Correlation analysis was performed using Spearman’s rank correlation coefficient (rho). Multivariate modelling was conducted using robust linear regression with bootstrapping (2000 replicates) to assess independent predictors of continuous outcomes, accounting for potential deviations from normal distribution.

A two-sided *p*-value < 0.05 was considered statistically significant for all analyses. The strength of correlations (Spearman’s rho) was interpreted as follows: 0–0.3 (weak), 0.3–0.5 (moderate), and >0.5 (strong). Effect sizes for categorical associations (φ or Cramer’s V) were interpreted as follows: 0.10–0.29 (weak), 0.30–0.49 (moderate), and ≥0.50 (strong).

## 3. Results

### 3.1. Baseline Clinical Characteristics

The clinical and laboratory data for the 200 patients included in this study are summarised in Table 1. Data were assessed for normality using the Shapiro–Wilk test and were found to be non-normally distributed. Therefore, continuous variables are presented as median (interquartile range: Q1–Q3) [minimum–maximum values], and categorical variables are presented as percentages (%).

The median preoperative age of the patients was 55 (50–57) [39–57] years, and 100% of the study participants were male. The median Body Mass Index (BMI) was 28 (27.2–28.6) [21.8–31] kg/m^2^. Baseline haemodynamics included a median systolic blood pressure (BP) of 139 (137–140) [130–156] mmHg, diastolic BP of 61 (53–66) [40–90] mmHg, and heart rate (HR) of 58 (54–64) [39–77] beats per minute (bpm).

Regarding co-morbidities and risk factors, 94.5% were smokers (current or within the last year), 74% had diabetes mellitus, and 100% presented with angina pectoris (NYHA Functional Class I–II). The median fasting glucose level was 4.9 (4.6–5.0) [4.0–5.8] mmol/L. Lipid profile analysis showed median Total Cholesterol (TC) of 3.5 (1.7–4.0) [0.8–5.0] mmol/L, High-Density Lipoprotein Cholesterol (HDL-C) of 1.2 (1.0–1.4) [0.8–1.7] mmol/L, Low-Density Lipoprotein Cholesterol (LDL-C) of 2.4 (2.2–3.4) [1.6–3.9] mmol/L, and Triglycerides (TG) of 2.1 (1.7–2.7) [1.1–3.1] mmol/L. The atherogenic index was 47.5% (interpretation/calculation method not specified).

Medication use prior to surgery included 25% adhering to a prescribed diet, 86.5% taking lipid-lowering drugs, 70.5% taking antihypertensive drugs, 86.5% taking glucose-lowering drugs, 71.5% taking anticoagulants, and 71% taking antiplatelet agents.

Preoperative echocardiography yielded a median Left Ventricular End-Diastolic Volume (LVEDV) (Simpson’s biplane) of 134 (128–138) [124–142] mL, and a median Left Ventricular Ejection Fraction (LVEF) (Simpson’s biplane) of 43 (41–45) [39–47] %. The baseline median plasma MMP9 level was 249 (222–275) [126–300] ng/mL.

Analysis of categorical variables revealed statistically significant associations between patients’ key clinical parameters. The strongest association was observed between diabetes mellitus and the use of hypoglycemic medications (φ = 0.37, *p* < 0.001), confirming appropriate therapeutic management. A moderate yet significant correlation emerged between smoking and elevated atherogenic index (φ = 0.25, *p* = 0.002), consistent with current understanding of smoking’s pathogenic role in dyslipidaemia development. A weak but significant association (V = 0.21, *p* = 0.03) between family history of cardiovascular disease and a high atherogenic profile underscores the importance of hereditary factors in cardiovascular risk assessment.

Notably, no significant association was found between smoking and diabetes mellitus (*p =* 0.15), suggesting that these risk factors may operate independently in this study population. The hypothesised link between family history and anticoagulant use also remained unconfirmed (*p =* 0.22), warranting further investigation in larger cohorts. All associations were adjusted for potential confounders, including age, sex, and BMI. Effect sizes were interpreted as follows: φ = 0.10–0.29 (weak), 0.30–0.49 (moderate), ≥0.50 (strong).

### 3.2. MMP9 Levels in the Control Group

For comparison, in the control group, in patients without signs of CVD, without coronary artery atherosclerosis, plasma MMP9 concentration averaged 89 ng/mL (78–95) [69–100] ng/mL, LVEF 64% (59–66) [54–77], LVEDV 138 mL (134–139) [110–146]. A statistically significant inverse correlation was found between LVEDV and the presence of diabetes mellitus (r = −0.374; p = 0.008), which may indicate a negative effect of hyperglycemia on left ventricular remodelling even in patients without obvious cardiac pathology. These data emphasise the importance of controlling cardiometabolic risk factors, in particular diabetes mellitus, even in individuals without diagnosed heart failure.

### 3.3. Association Between Preoperative Plasma MMP9 Concentrations and Baseline Characteristics

The median preoperative plasma MMP9 level in the patient group was 249 (222–275) [126–300] ng/mL. A significant, weak negative correlation was identified between preoperative MMP9 levels and baseline systolic blood pressure (Spearman’s rho = −0.322, *p* = 0.024). Additionally, a significant, weak negative correlation was found between MMP9 levels at 48 h postoperatively and preoperative baseline glucose levels (Spearman’s rho = −0.307, *p* = 0.032). No significant correlations were observed between preoperative MMP9 levels and age, LVEF, LVEDV, or other baseline laboratory parameters listed in Table 1.

### 3.4. Association Between Postoperative MMP9 Levels and Echocardiographic Parameters

**Figure 1:**
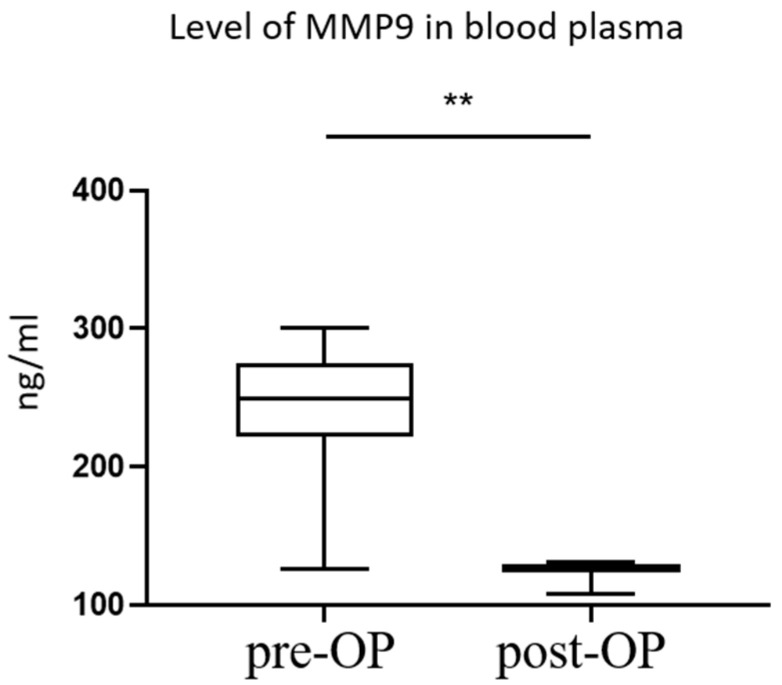
Preoperative and postoperative plasma MMP9 levels in patients. Box plots illustrate the distribution of plasma MMP9 concentrations measured preoperatively (Pre-OP) and 48 h postoperatively (Post-OP). Within each box plot, the central horizontal line indicates the median value. The box itself represents the interquartile range (IQR), extending from the first quartile (Q1, bottom edge of the box) to the third quartile (Q3, top edge of the box). The whiskers extend vertically from the box to show the minimum and maximum observed values in the dataset: Pre-OP Group: Median = 249 ng/mL; Q1 = 222 ng/mL; Q3 = 275 ng/mL; Minimum = 126 ng/mL; Maximum = 300 ng/mL. Post-OP Group: Median = 126 ng/mL; Q1 = 123 ng/mL; Q3 = 129 ng/mL; Minimum = 108 ng/mL; Maximum = 131 ng/mL. The difference between preoperative and postoperative MMP9 levels was statistically significant (assessed via paired Wilcoxon signed-rank test; ** *p* < 0.01).

Postoperative echocardiographic data for all patients included in this study are presented in Table 2. Circulating plasma MMP9 levels were significantly lower at 48 h postoperatively compared to preoperative levels. The median postoperative MMP9 concentration at 48 h was 126 (123–129) [108–131] ng/mL. This represents an approximately twofold reduction in MMP9 levels (*p* < 0.0001) (Figure 1).

Analysis of echocardiographic parameters in the early postoperative period indicated that myocardial revascularisation led to a significant improvement in LV systolic function. Concurrently, patients reported resolution of angina symptoms and a reduction in the severity of heart failure symptoms.

### 3.5. Association Between MMP9 Levels and Echocardiographic Parameters at Long-Term Follow-Up (1 Year)

Postoperative data, including echocardiographic parameters and plasma MMP9 levels obtained at 1 year following surgery, were analysed. Associations between plasma MMP9 concentrations and echocardiographic parameters at this time point were assessed using Spearman’s rank correlation coefficient.

A statistically significant negative correlation was identified between plasma MMP9 levels and Left Ventricular End-Diastolic Volume (LVEDV) at one-year post-surgery (Spearman’s r = −0.242, *p* = 0.01). This finding indicates an inverse relationship; higher MMP9 levels at one year were associated with lower LVEDV at the same time point, suggesting a potential role for MMP9 in myocardial remodelling during the late postoperative period.

Furthermore, a stronger negative correlation was found between 1-year MMP9 levels and Left Ventricular Ejection Fraction (LVEF) at one year (Spearman’s r = −0.625, *p* < 0.001). This indicates that elevated MMP9 levels at one-year are strongly associated with poorer LV systolic function, as measured via LVEF.

Multiple linear regression was used to assess the influence of MMP9, EF, and LVEDV on cardiometabolic parameters. The analysis revealed statistically significant associations; LVEDV was independently associated with presence of diabetes mellitus (β = 0.191, *p* = 0.014), elevated triglyceride levels (β = 0.333, *p* = 0.007), and NYHA functional class (β = 0.162, *p* = 0.039), explaining 13.1% of variability in these parameters (R^2^ = 0.131). Meanwhile, ejection fraction was a significant predictor of smoking status (β = −0.157, *p* = 0.049), accounting for 10.6% of variance (R^2^ = 0.106).

These findings suggest that increased LVEDV is associated with a more unfavourable cardiometabolic profile, whereas decreased LVEF is associated with smoking. Both parameters make an independent contribution to cardiovascular risk assessment.

Analysis comparing preoperative and 1-year postoperative MMP9 levels revealed a weak but statistically significant positive correlation between these two time points (Spearman’s r = 0.182, *p* = 0.01). This suggests some degree of tracking or persistence of relative MMP9 levels over the year following surgery, although the correlation is weak.

A significant negative correlation was also found between preoperative LVEDV and LVEDV measured at one-year post-surgery (Spearman’s r = −0.196, *p* = 0.005). This indicates that higher preoperative LVEDV was weakly associated with lower LVEDV at the 1-year follow-up. This correlation underscores the potential predictive value of baseline cardiac geometry for long-term structural outcomes following surgical treatment.

Finally, a statistically significant correlation was reported between preoperative LVEF and the control group (Spearman’s r = 0.364, *p* = 0.009), which was interpreted in the original analysis as potentially indicating that baseline LVEF might predict functional outcomes post-intervention.

**Figure 2:**
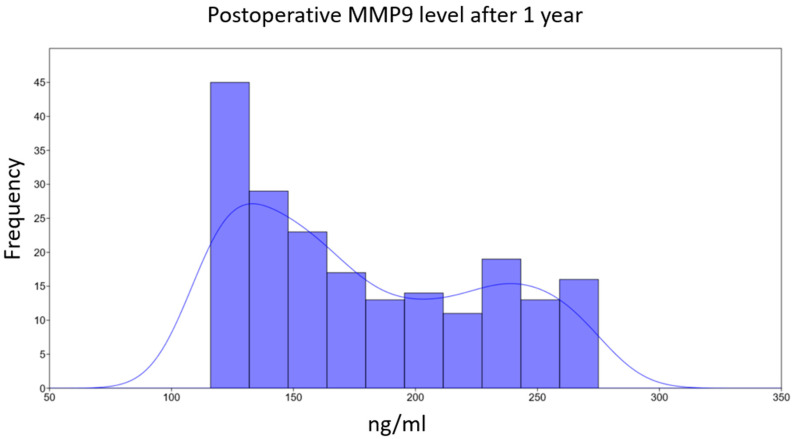
Frequency distribution of plasma MMP9 levels at 1-year follow-up, illustrating patient stratification into two subgroups. This histogram displays the distribution of plasma MMP9 concentrations measured in patients one year postoperatively. The *x*-axis represents plasma MMP9 concentration (in ng/mL), and the *y*-axis indicates the frequency (number of patients) observed at these concentrations. Based on this distribution, patients were stratified into two subgroups for analysis: one subgroup comprising patients with MMP9 levels greater than 200 ng/mL, and the other subgroup comprising patients with MMP9 levels less than or equal to 200 ng/mL.

Median values for MMP9, LVEF, and LVEDV at the 1-year follow-up are presented in Table 2. Upon examining the distribution of 1-year MMP9 levels, patients appeared to cluster into two subgroups based on median values (Figure 2).

Analysis revealed distinct characteristics between the subgroups defined by the 1-year plasma MMP9 concentration threshold of 200 ng/mL:

The high MMP9 subgroup (defined as MMP9 > 200 ng/mL) had a median MMP9 level of 238 (223–257) [200–275] ng/mL. These patients exhibited a median LVEF of 45% (39–49) [36–55] and a median LVEDV of 135 mL (127–145) [122–153].

Conversely, the low MMP9 subgroup (defined as MMP9 ≤ 200 ng/mL) had a median MMP9 level of 142 (120–163) [116–198] ng/mL. These patients demonstrated a median LVEF of 54% (42–57) [36–61] and a median LVEDV of 141 mL (130–148) [122–153].

Note: the first number corresponds to the median of the distribution, the interquartile range (Q1–Q3) is indicated in parentheses, and the minimum and maximum values of the indices are indicated in square brackets. Statistically significant differences were found in the criterion of LVEF (*p* = 0.023) and LVEDV (*p* < 0.001) levels depending on the indicators of MMP9 subgroups (*p* < 0.001). For other clinical parameters, no significant difference was found in groups according to MMP9 levels.

Further correlational analyses were performed, seemingly focusing primarily on the subgroup with elevated MMP9 levels:

Within the high MMP9 subgroup, a negative correlation was observed between MMP9 levels and LVEDV (Spearman’s r = −0.235, *p* = 0.008). This suggests that within this subgroup exhibiting high MMP9 overall, relatively higher MMP9 levels were associated with smaller LV end-diastolic volumes at the 1-year mark.

Additionally, a strong negative correlation was found between MMP9 levels and LVEF within this (with high MMP9) subgroup (Spearman’s r = −0.762, *p* < 0.001). This strong relationship indicates that higher MMP9 levels were closely associated with poorer LV systolic function (lower LVEF) in these patients one year after surgery. This finding may imply a potential role for sustained MMP9 elevation in the pathophysiology of adverse myocardial remodelling and dysfunction.

**Figure 3:**
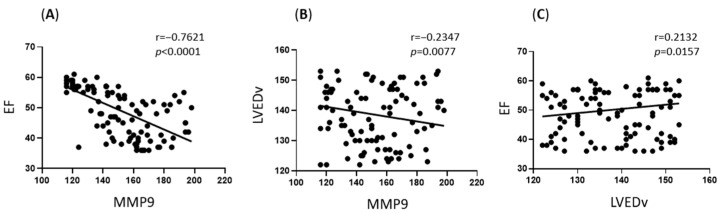
Correlation between plasma MMP9 levels (ng/mL) and echocardiographic parameters (LVEDV and LVEF) at 1-year follow-up. This figure illustrates the relationships between plasma MMP9 concentration and key echocardiographic parameters: Left Ventricular End-Diastolic Volume (LVEDV) and Left Ventricular Ejection Fraction (LVEF), measured one year postoperatively. Each point represents an individual patient. Solid lines indicate the linear trend. Associations were assessed using Spearman’s rank correlation coefficient (rho). (A) Correlation between 1-year plasma MMP9 concentration (*y*-axis, ng/mL) and 1-year Left Ventricular Ejection Fraction (LVEF, *x*-axis, %): Spearman’s rho = −0.762, *p* < 0.001. (B) Correlation between 1-year plasma MMP9 concentration (*y*-axis, ng/mL) and 1-year Left Ventricular End-Diastolic Volume (LVEDV, *x*-axis, mL): Spearman’s rho = −0.235, *p* = 0.008. (C) Correlation between 1-year Left Ventricular Ejection Fraction (LVEF, *y*-axis, %) and 1-year Left Ventricular End-Diastolic Volume (LVEDV, *x*-axis, mL): Spearman’s rho = 0.213, *p* = 0.016.

Interestingly, also within this patient subgroup (with high MMP9), LVEDV demonstrated a positive correlation with LVEF (Spearman’s r = 0.213, *p* = 0.016) (Figure 3). This positive association between volume and ejection fraction might reflect complex compensatory mechanisms or specific patterns of remodelling within this subset of patients.

## 4. Discussion

Despite haemodynamic parameters and quality of life often remaining satisfactory for a considerable period following surgical intervention, myocardial remodelling frequently continues due to the progression of fibrosis. This can contribute to further dilatation of the left ventricular cavity, a decline in LVEF, and consequently, a worsening of CHF. Given this, identifying predictors for long-term LV remodelling progression after surgical revascularisation becomes highly relevant, underscoring the necessity for closer monitoring to prevent irreversible consequences of CHF and to inform further management strategies [13].

Over recent years, biomarkers have been increasingly utilised as adjunctive indicators for risk stratification and disease prognosis. Correspondingly, MMPs have been widely investigated as potential markers for predicting LV remodelling following MI and the subsequent development of heart failure [14].

The MMP family comprises over 25 isoforms of zinc-dependent proteases that degrade the extracellular matrix (ECM) and are integral to tissue remodelling. Our focus is on MMP9 (gelatinase B), an extensively studied protease implicated in pathological remodelling. MMP9 is secreted by numerous cell types, including cardiomyocytes, endothelial cells, neutrophils, macrophages, and fibroblasts [15,16,17,18].

Blankenberg et al. were among the first to investigate MMP9 as a prognostic biomarker for LV dysfunction and long-term survival. In 1127 patients with confirmed coronary artery disease, higher baseline plasma MMP9 concentrations were associated with increased cardiovascular mortality over a mean follow-up of 4.1 years (62.2 ng/mL in fatal events vs. 47.8 ng/mL in survivors; *p* < 0.0001) [19]. Squire et al. demonstrated that elevated MMP9 levels post-MI are associated with larger LV volumes and dysfunction [20]. Inokubo et al. showed elevated MMP9 levels in acute coronary syndrome (ACS), suggesting active plaque rupture [21].

These studies, among others [22,23,24,25], support our initial findings: preoperative MMP9 levels were elevated in our patients with stenosed coronary arteries, reduced LVEF, and increased LV volumes, aligning with MMP9′s role in ischaemic myocardial remodelling. Following revascularisation, we observed a significant early (48 h) decrease in MMP9 concentrations. This biochemical change, accompanied by improved LVEF, suggests early favourable effects. Previous studies indicate that in IHD, processes like remodelling, inflammation, and oxidative stress elevate certain cytokines, activating MMPs. Conversely, eliminating myocardial ischaemia can attenuate these mechanisms, leading to reduced MMP levels [22,23,24,25].

Our 1-year follow-up data, however, revealed heterogeneous MMP9 responses, leading to patient stratification. The subgroup with persistently high 1-year plasma MMP9 concentrations (*p* < 0.001 vs. low group) exhibited less favourable long-term LVEF. This sustained MMP9 elevation might indicate ongoing adverse myocardial processes or persistent low-grade inflammation despite revascularisation, potentially contributing to CHF progression. It is noteworthy that higher 1-year MMP9 levels correlated with both lower LVEF and, somewhat counterintuitively, lower LVEDV. This observation may reflect that, within the 1-year post-revascularisation period and with patients generally receiving optimal medical therapy, the average LVEF in the high MMP9 group (median 45%) had not yet declined to a point that would consistently trigger the significant compensatory LV dilatation characteristic of more advanced or congestive heart failure.

The role of MMP9 is complex. Kelly et al. found that higher plateau MMP9 levels immediately post-AMI were paradoxically linked to less adverse remodelling and preserved LV function [26], suggesting that the timing, magnitude, and duration of MMP9 elevation are critical. Nevertheless, detrimental effects *of* sustained or excessively high MMP9 activity are supported by studies linking it to infarct expansion, LV wall thinning/rupture, cavity dilatation, and reduced LVEF [20,27,28], aligning with our 1-year findings.

Conversely, our low 1-year MMP9 subgroup exhibited more favourable dynamics, including better preservation of LV function. This might reflect a balanced interplay with endogenous tissue inhibitors of metalloproteinases (TIMPs) [29,30]. Therapeutic interventions may also contribute; statins can suppress MMP expression, partly by reducing vascular inflammation and promoting plaque stabilisation [23,31,32,33]. Furthermore, antihypertensive drugs like the calcium channel blocker lercanidipine have been shown to reduce MMP9 levels, possibly by mitigating oxidative stress, a key MMP9 activator [34,35,36].

## 5. Study Strengths and Limitations

Strengths: To our knowledge, this study is the first to demonstrate an association between distinct long-term (1-year) plasma MMP9 levels and parameters of LV function following off-pump myocardial revascularisation. This suggests MMP9 may serve as a potential biomarker for predicting long-term outcomes in this setting.

Limitations: Several limitations must be acknowledged. First, all patients in the surgical group were male, which limits the generalisability of our findings regarding gender. Second, before MMP9 could be considered for routine clinical use, larger multicentre studies are required, ideally comparing its prognostic value against or in combination with established biomarkers. Third, our MMP9 measurement frequency (preoperative, 48 h, 1 year) might not fully capture precise temporal dynamics; more frequent monitoring could offer greater insight. Finally, given that various medications can influence MMP9 levels, and lacking detailed data on long-term medication regimens and adherence, potential confounding effects of pharmacotherapy on 1-year MMP9 levels cannot be definitively excluded. The absence of a formal a priori sample size calculation is another limitation, although the achieved sample size provided sufficient data for the analyses performed.

## 6. Conclusions

Our findings suggest that plasma MMP9 levels, particularly those measured one year after off-pump CABG, are significantly associated with key indicators of left ventricular function. This indicates progress in identifying biomarkers for adverse myocardial remodelling post-revascularisation.

Looking forward, if further validated, MMP9 could potentially aid in risk stratification. For instance, persistently elevated MMP9 levels long after surgery might identify patients requiring closer monitoring or tailored secondary prevention strategies. While our study showed a weak link between preoperative MMP9 and some baseline factors, its utility as a preoperative decision-making tool for surgical candidacy requires much more robust investigation, focusing specifically on the independent predictive power of preoperative MMP9 for long-term clinical events and functional outcomes. Further research is warranted to confirm these findings in diverse populations, explore optimal MMP9 thresholds, and integrate MMP9 assessment into comprehensive risk prediction models.

## Figures and Tables

**Table 1:** Baseline clinical characteristics of the control group and patient group (preoperative).

*p*	Patient Preoperative Data	Control Group Data	Parameter
0.667	55 (50–57) [39–57]	56 (48–58) [49–59]	Age, years
0.001	100	78	Sex (Male), %
0.217	28 (27.2–28.6) [21.8–31]	27 (26–28) [28–31]	BMI (kg/m^2^)
<0.001	139 (137–140) [130–156]	137 (125–140) [111–150]	Systolic BP (mmHg)
0.012	61 (53–66) [40–90]	58 (54–65) [40–75]	Diastolic BP (mmHg)
0.016	58 (54–64) [39–77]	55 (52–58) [39–67]	Heart rate (bpm)
0.008	94.5	58	Smoking (current/last year), %
0.011	4.9 (4.6–5.0) [4.0–5.8]	4.8 (4.4–4.9) [4–5.8]	Glucose level (mmol/L)
0.016	74	64	Diabetes mellitus, %
1	100	100	Angina pectoris (NYHA Class I–II), %
0.473	3.5 (1.7–4.0) [0.8–5.0]	3.4 (1.7–3.50) [1.3–5]	Total Cholesterol (TC), mmol/L
0.223	1.2 (1.0–1.4) [0.8–1.7]	1.2 (1–1.3) [0.8–1.7]	HDL-C (mmol/L)
0.007	2.4 (2.2–3.4) [1.6–3.9]	2.2 (1.9–3.1) [1.2–3.9]	LDL-C (mmol/L)
0.041	2.1 (1.7–2.7) [1.1–3.1]	2 (1.5–2.5) [1.1–3.1]	Triglycerides (TG), mmol/L
0.160	52	32	Atherogenic Index, %
0.008	25	34	Prescribed diet, %
0.031	86.5	62	Lipid-lowering medication use, %
<0.001	70.5	62	Antihypertensive medication use, %
0.125	86.5	58	Glucose-lowering medication use, %
<0.001	71.5	12	Anticoagulant use, %
<0.001	71	62	Antiplatelet use, %
0.397	134 (128–138) [124–142]	138 (134–139) [110–146]	LVEDV (Simpson’s biplane), mL
<0.001	43 (41–45) [39–47]	64 (59–66) [54–77]	LVEF (Simpson’s biplane), %
<0.001	249 (222–275) [126–300]	89 (78–95) [69–100]	MMP9, ng/mL

Abbreviations: BMI—Body Mass Index; BP—Blood Pressure; bpm—beats per minute; HDL-C—High-Density Lipoprotein Cholesterol; LDL-C—Low-Density Lipoprotein Cholesterol; LVEDV—Left Ventricular End-Diastolic Volume; LVEF—Left Ventricular Ejection Fraction; MMP9—Matrix Metalloproteinase 9; TC—Total Cholesterol; TG—Triglycerides.

**Table 2:** Comparison of echocardiographic parameters and plasma MMP9 Levels between the control group and patient group (early postoperatively and at 1-year follow-up).

Patient Group—1-Year Follow-Up (*n* = 200)	Patient Group—Early Postoperative (48 h) (*n* = 200)	Control Group (*n* = 50)	Parameter
			Echocardiographic Parameters
139 (130–148) [122–153] *p* = 0.160	124 (121–127) [117–130] *p* < 0.001	138 (134–139) [110–146]	LVEDV (Simpson’s biplane), mL
49 (41–57) [36–61]	54 (54–55) [52–56]	64 (59–66) [54–77]	LVEF (Simpson’s biplane), %
*p* < 0.001	*p* < 0.001		Biomarker
165 (136–224) [116–275] *p* < 0.001	126 (123–129) [108–131] *p* < 0.001	89 (78–95) [69–100]	Plasma MMP9, ng/mL

Notes: Data are presented as median (interquartile range: Q1–Q3) [minimum–maximum values]. Abbreviations: LVEDV—Left Ventricular End-Diastolic Volume; LVEF—Left Ventricular Ejection Fraction; MMP9—Matrix Metalloproteinase 9. *p* indicates the significance of the difference from the control group.

## Data Availability

Data are available on reasonable request. The data underlying this article cannot be shared publicly to protect the privacy of individuals who participated in this study. The anonymised data may be shared on reasonable request to the corresponding author.
